# A draft genome assembly of reef-building octocoral *Heliopora coerulea*

**DOI:** 10.1038/s41597-023-02291-z

**Published:** 2023-06-14

**Authors:** Jack Chi-Ho Ip, Ming-Hay Ho, Benny K. K. Chan, Jian-Wen Qiu

**Affiliations:** 1 Department of Biology, Hong Kong Baptist University, Kowloon Tong, Hong Kong; 2 Biodiversity Research Center, Academia Sinica, Taipei, Taiwan

**Keywords:** Molecular ecology, Genome

## Abstract

Coral reefs are under existential threat from climate change and anthropogenic impacts. Genomic studies have enhanced our knowledge of resilience and responses of some coral species to environmental stress, but reference genomes are lacking for many coral species. The blue coral *Heliopora* is the only reef-building octocoral genus and exhibits optimal growth at a temperature close to the bleaching threshold of scleractinian corals. Local and high-latitude expansions of *Heliopora coerulea* were reported in the last decade, but little is known about the molecular mechanisms underlying its thermal resistance. We generated a draft genome of *H. coerulea* with an assembled size of 429.9 Mb, scaffold N50 of 1.42 Mb and BUSCO completeness of 94.9%. The genome contains 239.1 Mb repetitive sequences, 27,108 protein coding genes, 6,225 lncRNAs, and 79 miRNAs. This reference genome provides a valuable resource for in-depth studies on the adaptive mechanisms of corals under climate change and the evolution of skeleton in cnidarian.

## Background & Summary

Coral reefs are one of the most diverse and productive ecosystems, which support more than one-quarter of marine life with less than 2% of the ocean floor^1^. In recent decades, reef-building corals are threatened by anthropogenic climate change such as ocean warming and acidification^2,3^, as well as local stressors such as overfishing, pollution, and coastal development^4–6^. The world has lost almost 50% coral coverage since the 1950s^7^. With projected continued degradation of coral reefs, 90% of coral reefs may disappear in the next few decades^8–10^.

**Fig. 1:**
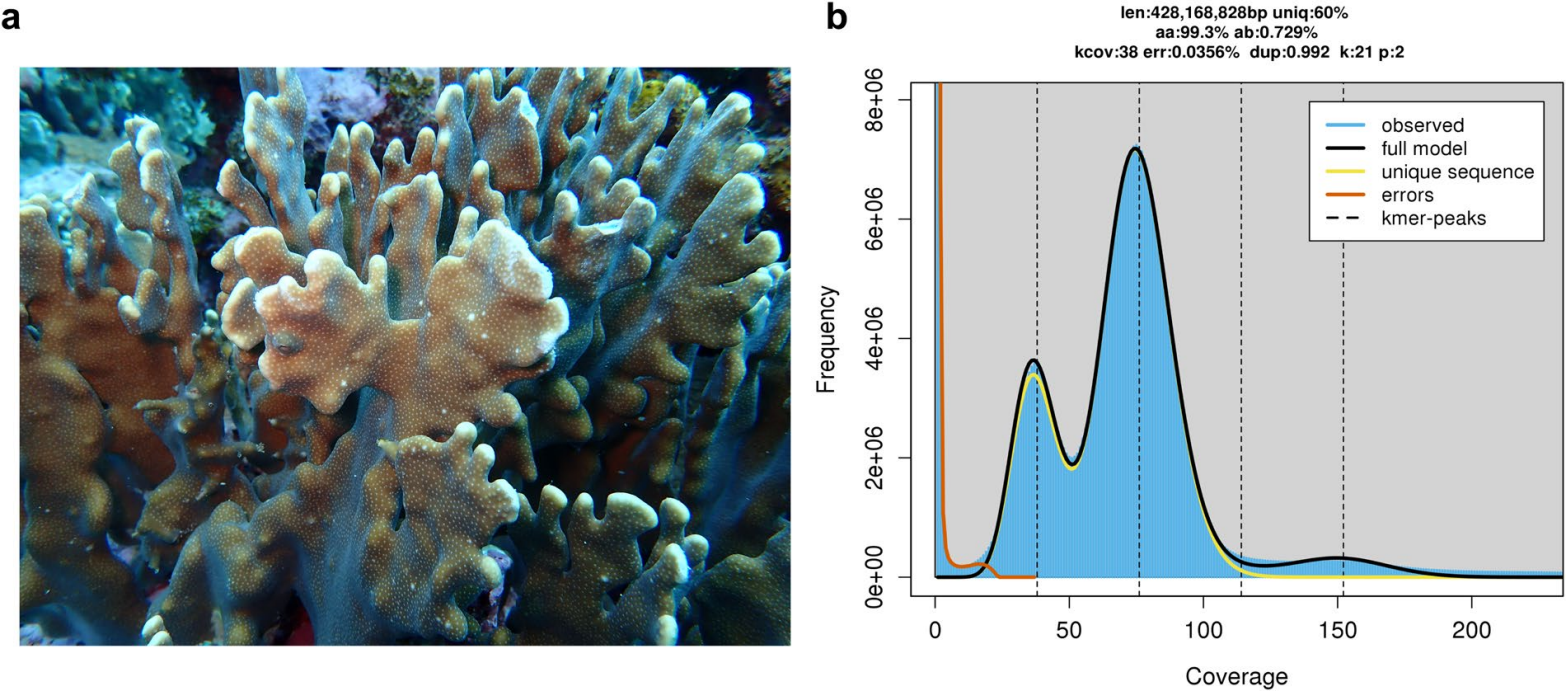
(**a**) A photograph of the blue coral *Heliopora coerulea* in the field (Photo credit: Benny K.K. Chan). (**b**) Kmer-21 histogram generated using Illumina reads. Genome size and heterozygosity rate were estimated using GenomeScope2^26^.

The blue corals (*Heliopora*) are the only genus of octocorals that form a massive hard skeleton and symbiosis with zooxanthellae like scleractinian corals^11^ (Fig. 1a). Due to their massive reef structure, blue corals are an important reef-building species in the Indo-West Pacific^11–14^. *H. coerulea*, with a characteristic blue skeleton, had long been regarded as the only extant member of the family Helioporidae, until the recent description of *H. hiberniana* (with white skeleton) in northwestern Australia^15^. Recent studies based on RAD-seq and Genotyping by sequencing in blue corals revealed there are also two distinct lineages of *H. coerulea* in the Kuroshio Current region^16,17^. Based on fossil records, the genus *Heliopora* were once widely distributed throughout the warm shallow oceans in the early Cretaceous^11,18^ (<120 million years ago, MYA). To date, *H. coerulea* is distributed in the shallow warm waters of the Indo-Pacific oceans^11,17^.

*Heliopora coerulea* is known to survive through bleaching events better than most scleractinian corals^15,19,20^. Recently, this species has been reported to expand from the tropics to the high-latitude Tsukazaki, Japan^21^. A shift of dominant taxa from scleractinian corals to *H. coerulea* has been reported in reefs of Ishigaki island, Japan^22^ and the South China Sea side of the Philippines^14,23^. In addition, laboratory experiments showed that *H. coerulea* had a higher growth rate when exposed at 31 °C – a temperature that would usually trigger the bleaching of scleractinian corals^7–9^ – than at 26 °C^24^.

**Fig. 2:**
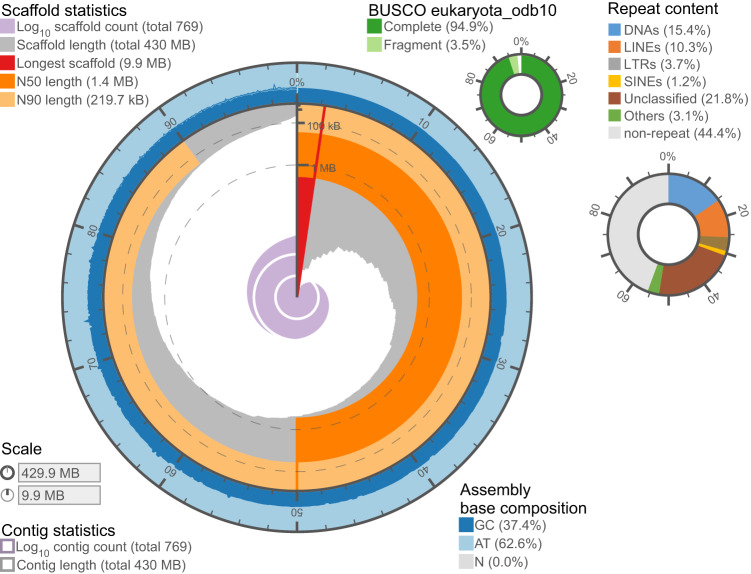
Snail plot visualization summarizing metrics of the *Heliopora coerulea* genome including the length of the longest contig (9.92 Mb; red line), N50 (1.42 Mb; dark orange), base composition, BUSCO completeness, and repeat content.

To facilitate molecular studies of blue corals to understand their thermal resistance, here, we report a draft genome assembly of *H. coerulea* generated using long-read PacBio HiFi sequencing (Tables 1, 2). The assembled genome size of *H. coerulea* is 429.9 Mb, consisting of 769 contigs with an N50 of 1.42 Mb, GC content of 37.4%, and 55.6% repeat elements (Fig. 2). The genome contains a total of 27,108 protein-coding genes with 95.7% functional annotated by BLASTp search against the published protein databases. In addition, RNA sequencing shows that the *H. coerulea* genome contains 6,225 lncRNAs and 79 miRNAs.

**Table 1:** A summary of *Heliopora coerulea* genome, mRNA, lncRNA, and miRNA sequencing data.

Sample	Library type	Sequencing platform	Raw data (million reads)	Filtered data (million reads)	Read length (bp)
**Genome**					
	350 bp insert size	Hiseq Xten	182.1 (54.6 Gb)	148.2 (42.0 Gb)	PE150
	500 bp insert size	Hiseq Xten	226.1 (67.8 Gb)	169.5 (46.8 Gb)	PE150
	PacBio HiFi	PacBio Sequal II	2.3 (31.8 Gb; N50 = 14.0 kb; mean length = 13.5 kb)		—
**mRNAseq**					
Replicate_1	cDNA	Hiseq Xten	48.4 (14.5 Gb)	28.0 (7.9 Gb)	PE150
Replicate_2	cDNA	Hiseq Xten	37.3 (11.2 Gb)	34.5 (9.5 Gb)	PE150
**lncRNAseq**					
Replicate_1	Long non-coding RNA	NovaSeq 6000	40.3 (12.0 Gb)	33.3 (9.3 Gb)	PE150
Replicate_2	Long non-coding RNA	NovaSeq 6000	40.4 (12.1 Gb)	34.5 (9.7 Gb)	PE150
**miRNA**					
Replicate_1	Micro RNA	NovaSeq 6000	11.6 (592.3 Mb)	11.2 (299.4 Mb)	SE50
Replicate_2	Micro RNA	NovaSeq 6000	12.6 (644.4 Mb)	11,7 (300.7 Mb)	SE50

**Table 2:** Statisitcs of assembled genome after filtering with binning, BLAST, and heterozygous contigs.

Items	Initial assembly	MetaBAT2	BLASTn	Purge Haplotigs
Genome size (Mb)	1309.7	600.2	586.0	428.2
No. of contig	12,153	2,364	2,248	769
N50 (Mb)	0.12	0.78	0.79	1.42
Longest contig (Mb)	10.11	9.92	9.92	9.92
Average length (Mb)	0.11	0.25	0.26	0.56
BUSCO eukaryota_odb10	C:96.0%, F:3.1%, M:0.9%	C:95.3%, F:3.1%, M:1.6%	C:95.3%, F:3.1%, M:1.6%	C:94.9%, F:3.5%, M:1.6%
BUSCO metazoa_odb10	C:90.1%, F:5.2%, M:4.7%	C:89.2%, F:5.1%, M:5.7%	C:89.2%, F:5.1%, M:5.7%	C:88.9%, F:5.5%, M:5.6%

For BUSCO score, C: number of complete BUSCOs; F, number of fragmented BUSCOs; M, number of missing BUSCOs.

## Methods

### Sample collection

The blue coral was collected by SCUBA at 5 m depth from Green Island, Taiwan (22°40′37′′N 121°28′23′′E) in April 2018. Coral fragments were transported in seawater to Biodiversity Research Center, Academia Sinica, Taipei, where they were kept in a 5 L aerated aquarium. To avoid contamination by bacteria or algae in the water, the coral fragments were rinsed several times in Milli-Q water immediately prior to DNA and RNA sampling. Coral fragments were immediately fixed in liquid nitrogen for DNA extraction and genome sequencing, whilst tissues were fixed in RNA*later* (Invitrogen, CA, USA) for RNA sequencing. All samples were stored at −80 °C in a freezer until subjected to extraction.

### Genomic sequencing

Genomic DNA was extracted from the coral tissue using the CTAB method^25^. DNA quality and quantity was measured using agarose gel electrophoresis and a Qubit fluorometer (Thermo Fisher Scientific, MA, USA), respectively. DNA samples were submitted to Novogene (Beijing, China) for library preparation and whole genome sequencing (Table 1). Briefly, 1 µg DNA was used to construct two libraries with 350-bp and 500-bp insert sizes using the NEBNext DNA Library Prep Kit (New England Biolabs, MA, USA), and sequenced on an Illumina HiSeq X Ten sequencer to generate 122.4 Gb paired-end reads with a read length of 150 bp. In addition, 10 µg DNA was used to construct a HiFi SMRTbell library using the SMRTbell Express Template Prep Kit 2.0, and sequenced on a PacBio Sequel II sequencer. Total of 31.8 Gb high-quality HiFi reads were produced using the circular consensus sequencing (CCS) mode on the PacBio long-read platform.

### RNA sequencing

Total RNA was extracted from the coral tissue using TRIzol reagent (Thermo Fisher Scientific, MA, USA) by following the manufacturer’s protocol. The quality of the RNA samples was determined with agarose gel electrophoresis and the quantity was determined using a Qubit fluorometer (Thermo Fisher Scientific, MA, USA). RNA samples were submitted to Novogene (Beijing, China) for mRNA, long non-coding RNA (lncRNA), and microRNA (miRNA) sequencing (Table 1). mRNA library was constructed using Illumina NEBNext Ultra RNA Library Prep Kit (New England Biolabs, MA, USA) and sequenced using an Illumina HiSeq X Ten sequencer to produce 150-bp paired-end reads. For lncRNA, ribosomal RNA was depleted from total RNA using Epicentre Ribo-Zero rRNA Removal Kit (Epicentre, WI, USA). The cDNA libraries were prepared using the NEBNext Ultra RNA Library Prep Kit (New England Biolabs, MA, USA), and sequenced on an Illumina NovaSeq platform under the paired-end mode to produce 150-bp reads. In addition, miRNA libraries were prepared using the NEBNext Multiplex Small RNA Library Prep Kit (Illumina, CA, USA) and sequenced on an Illumina NovaSeq platform to produce 50-bp single-end reads.

### Estimation of genome size

The genome size of *H. coerulea* was estimated using GenomeScope v2.0 with Illumina data^26^. Adaptors and low-quality reads (quality score <30, length <40 bp) of the Illumina data were trimmed with Trimmomatic v0.38^27^. To eliminate the zooxanthellae and prokaryotic reads, Illumina data were further filtered using bbmap.sh v39.01 (https://sourceforge.net/projects/bbmap/) against the Symbiodiniaceae genomes (*Symbiodinium minutum*, *S. microadriaticum*, *S. kawagutii*, and *S goreaui*) from ReefGenomics database (http://reefgenomics.org/) and NCBI Prokaryotic Refseq genomes with default settings. A total of 88.7 Gb Illumina reads were returned after quality filtering, and 77.9 Gb (87.8%) of them were from coral host. The clean Illumina data were used to generate a 21-kmer histogram using jellyfish v2.2.0^28^, and then characterized using GenomeScope v2.0, which predicted the genome size of 428.2 Mb and heterozygosity of 0.73% at a k-mer size of 21 (Fig. 1b).

### Genome assembly

*De novo* assembly of HiFi reads (N50 of 14.0 kb and mean length of 13.5 kb; Table 1) were performed using nextDenovo v2.5.0 (https://github.com/Nextomics/NextDenovo) under default settings. Algal and microbial sequences were removed by binning genome assembly with MetaBAT2 v2.15^29^, and BLASTn v2.11.0 + search against the 14 cnidarian genomes in Table 4, four Symbiodiniaceae genomes from ReefGenomics database (http://reefgenomics.org/), and NCBI Prokaryotic Refseq genomes with an E-value threshold of 1e-20. The initial assembly generated 1,309.7 Mb metagenome sequences (Table 2). After binning, a total of 170 bins were identified and the “Bin167” with 600.2 Mb and >100X coverage of Illumina data was selected (Table 2 and S1). BLASTn analysis filtered the potential symbiont sequence and resulted in the 586.0 Mb genome with 2,248 contigs. Possible alternative heterozygous contigs were further eliminated using Purge Haplotigs v1.1.230^30^ (Table 2). The completeness of the final genome assembly was assessed by analyzing the Benchmarking Universal Single-Copy Orthologs (BUSCO) v5.4.5 scores against the databases eukaryota_odb10 and eukaryota_odb10 under the genome mode^31^. QUAST v5.2 was used to assess the assembly statistics^32^. The total assembled size of the genome is 429.9 Mb in length and the N50 is 1.42 Mb (Table 3; Fig. 2).

**Table 3:** Genome assembly and annotation statistics of *Heliopora coerulea*.

Item	Number
**Genome assembly**	
Estimated genome size (Mb)	428.2
Assembly total length (Mb)	429.9
Repeat content (Mb)	239.1 (55.62%)
GC content (%)	37.4
No. of contigs	769
N50 (Mb)	1.42
Average length (Mb)	0.56
Max. length (Mb)	9.92
Min. length (kb)	17.9
No. of contig > 100 Kb	588
Genome coverage – PacBio HiFi	99.9%
Genome coverage – Illumina data	94.8%
Mapping rate – PacBio HiFi	91.4%
Mapping rate – Illumina data	88.4%
BUSCO eukaryota_odb10	C:94.9%, F:3.5%, M:1.6%
BUSCO metazoa_odb10	C:88.9%, F:5.5%, M:5.6%
**Genome annotation**	
Protein coding genes	27,108
Average gene length (bp)	1,754
With annotation	25,955 (95.7%)
BUSCO eukaryota_odb10	C:95.7%, F:2.7%, M:1.6%
BUSCO metazoa_odb10	C:92.4%, F:2.9%, M:4.7%

For BUSCO score, C: number of complete BUSCOs; F, number of fragmented BUSCOs; M, number of missing BUSCOs.

**Fig. 3:**
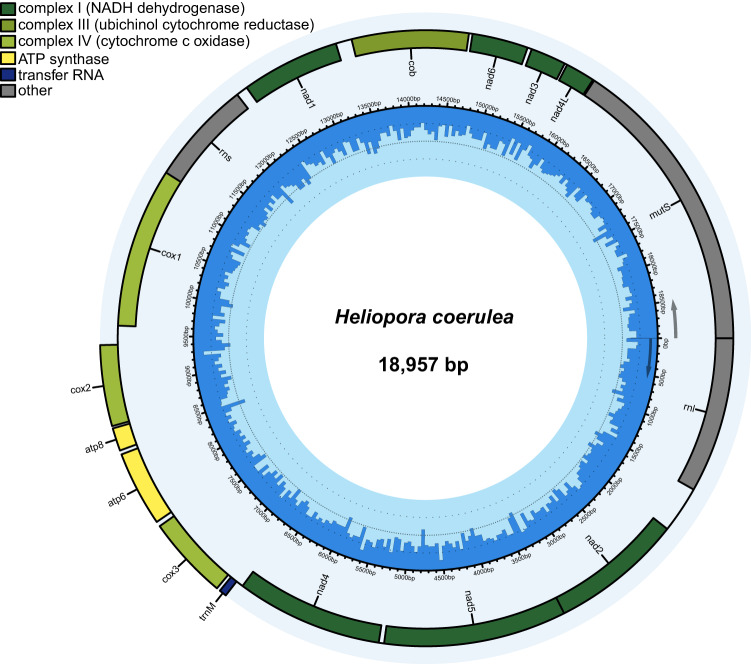
Mitogenome map of *Heliopora coerulea*. The outer circle shows the genes with the plus strand inside and minus strand outside. The GC content is plotted in the second inner circle at 50-bp sliding windows, depicted in dark blue.

In addition, the mitogenome of *H. coerulea* was assembled with Illumina clean reads using Norgal v1.0 under the default settings^33^, and annotated using MITOS2 online^34^ and tBLASTn v2.11.0 + search against the published *H. coerulea* MT genome (GenBank: OL616236). The *H. coerulea* mitogenome is 18,957 bp in length with 14 protein-coding genes (Fig. 3), which is 100% identical with OL616236 in GenBank.

### mRNA annotation

The protein coding genes of the *H. coerulea* genome were predicted using MAKER v3.0 pipeline^35^ according to Ip *et al*.^36^. In brief, repeat contents in the genome were identified using RepeatMasker v4.1.2-p1 (http://www.repeatmasker.org/; settings: “-e rmblast -s -gff”) with RepBase library version 20181026^37^ and species-specific repeat libraries in RepeatModeler v2.0.3^38^ under the “LTRStruct” option and the default setting for other parameters. A total of 239.1 Mb (55.6%) of the *H. coerulea* genome consists of repetitive sequences, including 30.6% transposable elements, 21.8% unclassified repeats, and 3.1% simple repeats and low complexity sequences (Table 3 and Fig. 2).

Raw mRNA reads were trimmed using Trimmomatic v0.38^27^ (quality score <30, length <40 bp). The clean reads were *de novo* and genome-guided assembled using Trinity v2.5.1^39^ under the default settings. Cnidaria protein sequences from UniProt database were used as protein evidence. Augustus v3.4^40^ and SNAP v2006-07-28^41^ were used for *ab initio* gene prediction. All predicted gene models were integrated into a consensus weighted annotation with EVidenceModeler v1.1.1^42^ under the default settings in Maker3. In addition, PASA v2.4.1 was used to improve the Maker result using the *de novo* transcriptome^43^. Finally, we obtained 27,108 predicted protein-coding genes with an N50 of 1,754 bp (Table 3).

The BUSCO completeness of predicted gene models was assessed against eukaryota_odb10 and metazoa_odb10 datasets^31^ under the protein mode. The predicted genes were functionally annotated using Diamond v2.0.13.151 BLASTp^44^ against UniProt and Swissport databases under the “ultra-sensitive” option and an E-value threshold of 1e-5. Gene functional annotation was conducted using eggNOG-mapper v2^45^ for Gene Ontology (GO), Kyoto Encyclopedia of Genes and Genomes (KEGG) pathways and Pfam domain.

### lncRNA annotation

The raw lncRNA reads were filtered to remove adapter and low-quality reads (quality score <30, length <40 bp) using Trimmomatic v0.38^27^. The clean lncRNA reads were mapped to the *H. coerulea* genome using HISAT2 v2.1.0^46^ under the default settings. The resulting bam files were then assembled into transcript models using StringTie v1.3.4d^47^ under the default settings. The assembled transcripts were processed through FlExible Extraction of LncRNAs (FEELnc) v0.2.1^48^ for lncRNA identification and classification. Briefly, the script FEELnc filter.pl was used to remove transcripts with one exon, a size < 200 bp, and overlapping with predicted protein-coding regions. The coding potential score of each candidate transcript was calculated using the script FELLnc_codpot.pl under the shuffle mode. Finally, the FEELnc_classifier.pl was used to classify potential lncRNA with respect to the localization and the direction of transcription of nearby protein-coding genes. A total of 6,225 lncRNA genes were predicted in the *H. coerulea* genome (Tables S2, S3).

### miRNA annotation

miRNA analysis was conducted according to Ip *et al*.^36^. Briefly, raw miRNA reads were trimmed with fastp v0.20.0^49^ under the settings of length_required = 18, max_length = 35, unqualified_percent_limit = 30, n_base_limit = 0. The clean reads were then combined and mapped to the genome using the mapper.pl script in miRDeep2 v2.0.1.2^50^ using bowtie v1.2.2^51^. miRNAs were predicted using the miRDeep2.pl script in miRDeep2 with the Cnidaria mature miRNAs from miRBase v22.1^52^. The predicted miRNAs were filtered with a miRDeep2 score ≥ 4, star (complementary) and mature read count ≥ 5, and a significant Randfold *p*-value. The target genes of miRNAs were predicted using miRanda v3.3a^53^ with a miRanda score ≥ 140, a dimer binding free energy < −5 kcalmol^−1^, and strict 5′ seed pairing. In total, we detected 79 miRNA candidates ranging from 20 to 24 nt in length, and 10,636 mRNAs were predicted as their potential targets (Tables S4, S5).

### Phylogeny, divergence, and gene family analyses

**Fig. 4:**
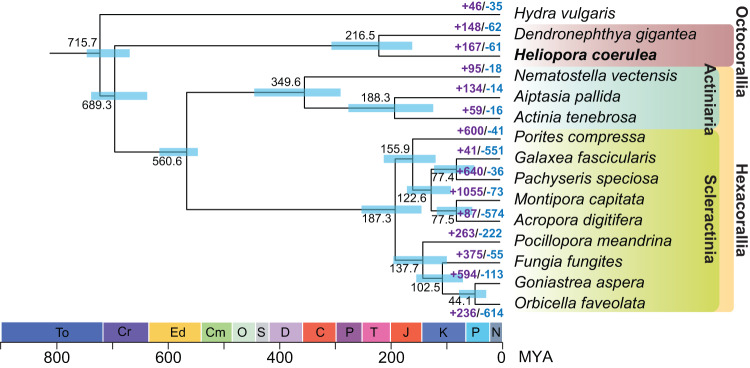
Maximum-likelihood phylogenomic tree with divergence time of *Heliopora coerulea* and other cnidarians. Bootstrap support is 100 at all nodes. Each blue line indicates a 95% confidence interval for a divergence time. Numbers on the branch show the lineage-specific expanded (+) and contracted (−) gene families (details in Table S7).

Orthologous groups among *H. coerulea* and 13 anthozoans with the outgroup species *Hydra vulgaris* (details in Table 4 and Table S6) were identified using OrthoFinder v2.5.4 under the “diamond_ultra_sens” option^54^. A total of 407 single-copy genes were aligned using MUSCLE v3.8.31^55^ and trimmed using TrimAL v1.4^56^. The aligned sequences with 91,426 amino acid positions and 1.1–13.9% gaps were concatenated for phylogenetic analysis using a maximum-likelihood method implemented in IQ-TREE v2.13^57^, with the best model of Q.insect + F + I + G4 and 1000 bootstrapping replicates. MCMCtree implemented in PAML v4.9h^58^ was used to estimate divergence times using the burn-in, sample frequency and number of samples of 10000000, 1000 and 10000, respectively. The node calibration among cnidarians was based on fossil records (i.e., ~55 MYA for *Acropora*^59^, ~145 MYA for Helioporacea^18^, ~540 MYA for Hexacorallia^60^) and TIMETREE database^61^ (i.e., Edwardsiidae for 280 – 490 MYA, Anthozoa for 520 – 740 MYA). Using the orthologous results, we performed the gene family expansion and contraction for each node using CAFÉ v4.2^62^. These analyses revealed that *H. coerulea* is sister to the soft coral *Dendronephthya gigantea*, which split during Triassic (~216 MYA, 95% confidence interval of 157–301 MYA; Fig. 4). This *D. gigantea* + *H. coerulea* clade is then sister to the Hexacorallia clade, consistent with a previous phylogenetic analysis of 234 anthozoans^63^. Gene family analysis detected 167 expanded and 61 contracted gene families in *H. coerulea* (Fig. 4; Table S7).

**Table 4:** Assembly statistics of 15 cnidarian genomes.

Species	Genome (Mb)	Scaffold No.	GC (%)	N50 (Mb)	Max (Mb)	Gene No.	BUSCO genome – eukaryota_odb10	BUSCO genome – metazoa_odb10	BUSCO gene – eukaryota_odb10	BUSCO gene – metazoa_odb10	Reference
*Hydra vulgaris*	819.4	56	26.9	77.98	55.05	32,703	C:96.5%[S:95.7%,D:0.8%] F:2.0%,M:1.5%,n:255	C:92.9%[S:92.3%,D:0.6%] F:3.0%,M:4.1%,n:954	C:99.2%[S:78.8%,D:20.4%] F:0.0%,M:0.8%,n:255	C:95.4%[S:74.5%,D:20.9%] F:0.9%,M:3.7%,n:954	GCF_022113875.1
*Dendronephthya gigantea*	286.2	1321	30.08	1.45	7.80	28,741	C:96.1%[S:84.7%,D:11.4%] F:2.4%,M:1.5%	C:88.7%[S:79.4%,D:9.3%] F:5.3%,M:6.0%	C:98.4%[S:74.1%,D:24.3%] F:0.4%,M:1.2%	C:95.2%[S:73.5%,D:21.7%] F:0.8%,M:4.0%	Jeon *et al*.^76^
*Heliopora coerulea*	429.9	769	37.4	1.42	9.92	27,108	C:94.9%[S:92.2%,D:2.7%] F:3.5%,M:1.6%,n:255	C:88.9%[S:86.2%,D:2.7%] F:5.5%,M:5.6%,n:954	C:95.7%[S:81.2%,D:14.5%] F:2.7%,M:1.6%,n:255	C:92.4%[S:80.0%,D:12.4%] F:2.9%,M:4.7%,n:954	This study
*Nematostella vectensis*	269.4	47	29.49	17.87	22.17	32,370	C:97.3%[S:97.3%,D:0.0%] F:2.7%,M:0.0%	C:94.6%[S:94.1%,D:0.5%] F:2.6%,M:2.8%	C:99.2%[S:74.1%,D:25.1%] F:0.0%,M:0.8%	C:97.7%[S:73.1%,D:24.6%] F:0.4%,M:1.9%	GCF_932526225.1
*Aiptasia pallida*	256.1	4312	24.31	0.44	1.84	27,753	C:94.9%[S:92.9%,D:2.0%] F:3.1%,M:2.0%	C:91.8%[S:89.3%,D:2.5%] F:4.3%,M:3.9%	C:96.1%[S:85.9%,D:10.2%] F:2.7%,M:1.2%	C:94.7%[S:83.5%,D:11.2%] F:2.1%,M:3.2%	GCA_001417965.1
*Actinia tenebrosa*	486.8	614	39.06	2.84	4.86	30,327	C:93.4%[S:91.4%,D:2.0%] F:4.3%,M:2.3%	C:93.0%[S:91.4%,D:1.6%] F:3.6%,M:3.4%	C:88.6%[S:85.9%,D:2.7%] F:5.9%,M:5.5%	C:88.6%[S:87.7%,D:0.9%] F:4.8%,M:6.6%	ReefGenomics
*Pocillopora meandrina*	376.6	212	38.03	10.02	21.65	31,840	C:98.8%[S:98.0%,D:0.8%] F:0.4%,M:0.8%	C:96.1%[S:94.9%,D:1.2%] F:2.0%,M:1.9%	C:96.5%[S:96.1%,D:0.4%] F:3.1%,M:0.4%	C:96.9%[S:95.5%,D:1.4%] F:1.8%,M:1.3%	Stephens *et al*.^77^
*Fungia fungites*	606.3	7424	33.38	0.32	1.80	38,209	C:92.2%[S:91.8%,D:0.4%] F:7.1%,M:0.7%	C:89.9%[S:89.3%,D:0.6%] F:5.8%,M:4.3%	C:86.7%[S:85.5%,D:1.2%] F:10.2%,M:3.1%	C:84.3%[S:83.0%,D:1.3%] F:9.7%,M:6.0%	ReefGenomics
*Goniastrea aspera*	764.9	5396	35.09	0.52	2.90	35,901	C:95.7%[S:95.7%,D:0.0%] F:3.5%,M:0.8%	C:93.2%[S:92.3%,D:0.9%] F:3.4%,M:3.4%	C:86.7%[S:86.3%,D:0.4%] F:9.0%,M:4.3%	C:85.4%[S:84.4%,D:1.0%] F:7.9%,M:6.7%	ReefGenomics
*Orbicella faveolata*	485.5	1933	22.81	1.16	4.77	32,587	C:85.5%[S:85.1%,D:0.4%] F:10.6%,M:3.9%	C:85.3%[S:84.6%,D:0.7%] F:8.5%,M:6.2%	C:87.4%[S:72.5%,D:14.9%] F:7.5%,M:5.1%	C:87.2%[S:72.7%,D:14.5%] F:6.6%,M:6.2%	GCF_002042975.1
*Porites compressa*	592.5	608	39.2	4.00	18.35	44,130	C:99.2%[S:98.0%,D:1.2%] F:0.4%,M:0.4%	C:95.9%[S:93.9%,D:2.0%] F:2.0%,M:2.1%	C:96.9%[S:94.9%,D:2.0%] F:2.4%,M:0.7%	C:95.8%[S:93.4%,D:2.4%] F:2.0%,M:2.2%	Stephens *et al*.^77^
*Pachyseris speciosa*	984.4	2368	39.56	0.77	4.62	39,160	C:95.6%[S:92.9%,D:2.7%] F:2.4%,M:2.0%	C:95.4%[S:91.8%,D:3.6%] F:1.6%,M:3.0%	C:86.7%[S:84.7%,D:2.0%] F:9.8%,M:3.5%	C:86.0%[S:83.5%,D:2.5%] F:6.4%,M:7.6%	ReefGenomics
*Galaxea fascicularis*	334.2	11269	38.56	0.09	0.87	22,418	C:88.6%[S:88.6%,D:0.0%] F:9.0%,M:2.4%	C:89.7%[S:89.3%,D:0.4%] F:5.9%,M:4.4%	C:85.5%[S:85.5%,D:0.0%] F:9.4%,M:5.1%	C:83.1%[S:82.6%,D:0.5%] F:9.0%,M:7.9%	ReefGenomics
*Acropora digitifera*	415.8	956	38.87	1.86	7.63	25,278	C:93.7%[S:92.9%,D:0.8%] F:4.3%,M:2.0%	C:92.8%[S:92.6%,D:0.2%] F:3.7%,M:3.5%	C:71.0%[S:65.1%,D:5.9%] F:18.4%,M:10.6%	C:74.6%[S:67.7%,D:6.9%] F:13.0%,M:12.4%	Shinzato *et al*.^78^
*Montipora capitata*	780.5	1699	39.65	47.72	48.53	54,384	C:99.2%[S:98.0%,D:1.2%] F:0.0%,M:0.8%	C:95.7%[S:94.0%,D:1.7%] F:2.2%,M:2.1%	C:96.5%[S:95.3%,D:1.2%] F:3.5%,M:0.0%	C:95.2%[S:92.9%,D:2.3%] F:3.2%,M:1.6%	Stephens *et al*.^77^

For BUSCO score, C: number of complete BUSCOs; S: number of Complete and single-copy BUSCOs, D: number of Complete and duplicated BUSCOs, F, number of fragmented BUSCOs; M, number of missing BUSCOs.

## Data Records

The Illumina, PacBio HiFi, and RNAseq data have been deposited in NCBI Sequence Read Archive with accession number SRR23530023^64^, SRR23530024^65^, SRR23530025^66^, SRR23530026^67^, SRR23530027^68^, SRR23530028^69^, SRR23530029^70^, SRR23530030^71^, and SRR23530031^72^, under Bioproject accession number PRJNA936655. The genome assembly has been deposited at GenBank with accession number JASJOG000000000^73^. The genome annotation (“Hco_maker_PASA_Final.gff”) and predicted genes (“Hco_v1.transcript.fasta” and “Hco_v1.protein.fasta”), lncRNA (“Hco_lncRNA.fasta”), and miRNA (“Hco_miRNA_mature.fasta”) has been deposited in the Figshare database^74^.

## Technical Validation

The quality of *H. coerulea* genome assembly was assessed by several approaches: (i) comparison with the estimated genome size, which is also ~430 Mb in total length (Figs. 1b, 2); (ii) obtaining the complete mitogenome, which is 100% identical in size and gene order with a published mitogenome of the same species (GenBank: OL616236; Fig. 3); (iii) conducting QUAST analysis, which showed that the assembly statistics of *H. coerulea* is comparable with published cnidarian genomes (Table 4); (iv) conducting BUSCO analysis, which identified 98.4% eukaryotic BUSCOs and 94.4% metazoan BUSCOs in the *H. coerulea* genome, and 98.4% eukaryotic BUSCOs and 95.3% metazoan BUSCOs in its predicted gene models (Table 4); (v) conducting the analysis of genome coverage using SAMtools v1.15.1^75^, which showed 100% genome coverage and 91.4% mapping rate of PacBio HiFi reads, and 94.8% genome coverage and 88.4% mapping rate of Illumina short reads (Table 3). These results indicated the *H. coerulea* assembly is of high-quality.

## Supplementary information

SUPPLEMENTARY INFORMATION

## Data Availability

All bioinformatic tools used in this study were executed according to the corresponding manual and protocols. The version and code and parameters of the main bioinformatic tools are described below. (1) Trimmomatic v0.38, parameters used: “PE -phred33 ILLUMINACLIP:TruSeq. 3-PE.fa:2:30:10 LEADING:3 TRAILING:3 SLIDINGWINDOW:4:30 MINLEN:40”. (2) jellyfish v2.2.0, parameters used: “-C -m 21”. (3) GenomeScope v.2.0, parameters used: ploidy 2 and kmer_length 21. (4) nextDenovo v2.5.0, parameters used: default. (5) Purge Haplotigs v1.1.2, parameters used: default. (5) MetaBAT v 2.12.1, parameters used: default. (6) BLASTn v2.11.0+, parameters used: “-evalue 1e-20 -max_target_seqs. 1”. (8) BUSCO v5.4.5, parameters used: lineage_dataset eukaryota_odb10 (255 BUSCOs) and metazoa_odb10 (954 BUSCOs). (9) Norgal v1.0, parameters used: default. (10) MAKER v3.0, parameters used: default. (11) RepeatMasker v4.1.2-p1, parameters used: “-e rmblast -s -gff”, Database: Dfam v3.1 and RepBaseRepeatMaskerEdition-20181026. (12) RepeatModeler v 2.0.3, parameters used: “-LTRStruct”. (13) Trinity v2.5.1, parameters used: default. (14) Augustus, version 3.4.0, parameters used: species = Database trained with BUSCO. (15) SNAP v2006-07-28, parameters used: default. (16) EVidenceModeler v1.1.1, parameters used: default settings in Maker3. (17) PASA v2.4.1, parameters used: “-C -R -T–ALIGNERS blat”. Augustus, version 3.4.0, parameters used: species = Database trained with BUSCO, alternatives-from-evidence = true, hintsfile = Output of RepeatMasker. (18) Diamond v2.0.13.151 BLASTp, parameters used: “-ultra-sensitive -max-target-seqs. 1 -evalue 1e-5”. (19) HISAT2 v2.1.0, parameters used: default. (20) StringTie v1.3.4d, parameters used: default. (21) FEELnc v0.2.1, parameters used: default. (22) fastp v0.20.0, parameters used: “length_required = 18, max_length = 35, unqualified_percent_limit = 30, n_base_limit = 0”. (23) miRDeep2 v2.0.1.2, parameters used: default. (24) miRanda v3.3a, parameters used: “-sc 140 -en -5 -strict”. (25) OrthoFinder v2.5.4, parameters used: “-S diamond_ultra_sens”. (26) IQ-TREE v2.1.3, parameters used: “-m TEST -bb 1000”. (27) MCMCtree implemented in PAML v4.9 h, parameters used: Tree topology from IQ-TREE result, fossil records in Fig. 4, burn-in: 10000000, sample frequency: 1000, and number of samples: 10000. (28) CAFÉ v4.2, parameters used: default. (29) QUAST v5.2, parameters used: default. (30) bbmap v39.01, parameters used: bbsplit.sh and mapPacBio.sh with default settings. (31) SAMtools v1.15.1, parameters used: command = coverage, depth, with default settings.
